# Effects of Medicinal Plant Extracts and Photosensitization on Aflatoxin Producing* Aspergillus flavus* (Raper and Fennell)

**DOI:** 10.1155/2017/5273893

**Published:** 2017-05-02

**Authors:** Loise M. Njoki, Sheila A. Okoth, Peter M. Wachira

**Affiliations:** School of Biological Sciences, University of Nairobi, P.O. Box 30197-00100, Nairobi, Kenya

## Abstract

This study was undertaken with an aim of exploring the effectiveness of medicinal plant extracts in the control of aflatoxin production. Antifungal properties, photosensitization, and phytochemical composition of aqueous and organic extracts of fruits from* Solanum aculeastrum*, bark from* Syzygium cordatum, *and leaves from* Prunus africana*,* Ocimum lamiifolium*,* Lippia kituiensis,* and* Spinacia oleracea* were tested. Spores from four-day-old cultures of previously identified toxigenic fungi, UONV017 and UONV003, were used. Disc diffusion and broth dilution methods were used to test the antifungal activity. The spores were suspended in 2 ml of each extract separately and treated with visible light (420 nm) for varying periods. Organic extracts displayed species and concentration dependent antifungal activity.* Solanum aculeastrum* had the highest zones of inhibition diameters in both strains: UONV017 (mean = 18.50 ± 0.71 mm) and UONV003 (mean = 11.92 ± 0.94 mm) at 600 mg/ml. Aqueous extracts had no antifungal activity because all diameters were below 8 mm.* Solanum aculeastrum* had the lowest minimum inhibitory concentration at 25 mg/ml against* A. flavus* UONV017. All the plant extracts in combination with light reduced the viability of fungal conidia compared with the controls without light, without extracts, and without both extracts and light. Six bioactive compounds were analyzed in the plant extracts. Medicinal plant extracts in this study can control conidia viability and hence with further development can control toxigenic fungal spread.

## 1. Introduction 

*Aspergillus flavus* is ubiquitous, saprophytic, and a weak parasite [1]. The fungus contaminates a wide range of cereals and nuts like maize, wheat, sorghum, and groundnuts, which serve as staple foods in most parts of Africa. Toxigenic* A. flavus* have been reported to contaminate these products and produce aflatoxins which are carcinogenic, mutagenic, and lethal fungal metabolites [2–4]. Aflatoxins have been classified as class 1 poisons by the International Agency for Research on Cancer (IARC) [5]. Aflatoxins also contaminate feed; hence products like meat, milk, cheese, and eggs get contaminated when animals consume the aflatoxin contaminated feed [6, 7]. Aflatoxin B1 is the major type of aflatoxins produced by* A. flavus* [3].* Aspergillus flavus* is the main fungi producing aflatoxin [1].

Aflatoxicosis was first reported in Kenya in 1982 [8]. More outbreaks have since been recorded in 2001, 2004, 2006, 2008, and 2010 [9, 10]. Records of aflatoxin contamination in food and feed are widespread in tropical and subtropical regions where climatic conditions and storage practices favor growth of fungi [11]. Aflatoxin production is influenced by aeration, moisture, temperature, and substrate and the control methods under trials like biological [12], cultural, and chemical ones [13] are all based on manipulation of these factors. Many African countries are now in the process of including regulation framework within their food policies to help control exposure to aflatoxins [1].

Traditionally, many plants have been used successfully for medicinal purposes [4]. Aromatic substances in plants, specifically secondary metabolites like alkaloids, flavonoids, saponins, glycosides, and tannins, are able to protect plants from invaders such as fungi, bacteria, and nematodes [14]. According to World Health Organization (2001), 80% of African and Asian communities rely on traditional herbal medicines for primary healthcare. This is because herbal medicines are safer and cheaper compared to synthetic medicines [15, 16].

This study exploited the ability of known medicinal plant extracts to control the growth of* A. flavus* conidia. Photosensitization has also been reported to kill both metabolically active and dormant structures such as conidia, unlike conventional fungicides that kill only metabolically active cells [17]. It involves hitting of a photosensitizer by light of a specific wavelength, which makes the photosensitizer reactive thereby killing the toxigenic cells [18]. The technique has been reported as a safe and a potential control of mycotoxigenic fungi [19]. However, very few photosensitizers have been approved clinically for use against toxigenic microbes, hence needing identification of safe photosensitizers. Plant extracts, on the other hand, are biodegradable and hence environmentally friendly [20]. The aim of this study is to determine the antifungal activity and phytochemical composition of the plants extracts. The ability of visible light to stimulate the bioactive compounds in the plant extracts (photosensitization) and hence increase in the antifungal activity of the extracts against toxigenic* A. flavus *which causes aflatoxin production will also be tested.

## 2. Materials and Methods

### 2.1. Collection of Plant Material

Five plants known for their medicinal value were collected from Gakoe forest in Gatundu district, Central Region of Kenya. These were* Ocimum lamiifolium *leaves (LMM 2015/05),* Prunus africana* leaves (LMM 2015/03),* Solanum aculeastrum *fruits (LMM 2015/01),* Lippia kituiensis *leaves (LMM 2015/04), and* Syzygium cordatum *bark (LMM 2015/02). Fresh leaves of* Spinacia oleracea* (LMM 2015/06) were also collected from the local market. The identity of the plants was confirmed using reference material from the University of Nairobi herbarium where voucher specimens were deposited.

### 2.2. Crude Plant Extract Preparation

The selected plant parts were air-dried at room temperature, chopped, and ground into powder. Dichloromethane-methanol (1 : 1) mixture was used for organic extraction. Two hundred and fifty grams of each ground extract was soaked in 1 L of the organic solvents for 48 hours. A rotary evaporator was used to filter and concentrate the organic extract, hence obtaining a semisolid residue for use [4]. Distilled water was used for aqueous extraction. Two hundred and fifty grams of each ground extract was soaked in 500 ml of distilled water in a glass beaker sealed with aluminum foil for five days. The extract was then filtered using Whatman number 1 filter paper. The filtrate was evaporated and dried using a freeze-drier to get powder [21]. The resulting products were stored at 4°C.

### 2.3. Preparation of Fungal Spore Suspension

Toxigenic* A. flavus* strains used in this study were obtained from the School of Biological Sciences Mycology Culture Collection. The isolates used were UONV017 and UONV003 and they had been tested for toxigenicity through molecular characterization according to [3]. The isolates were transferred from the stock cultures into sterile PDA plates and incubated for 4 days at 29°C. Spores were aseptically harvested and suspended in sterile distilled water with three drops of Tween 80 solution and standardized to a turbidity of 1 McFarland solution (3 × 10^8^ CFU/ml).

### 2.4. Determination of Inhibition Concentration

Antifungal activities of the plant extracts were evaluated using the disc diffusion as described by Sigei et al. [22] and according to National committee of clinical and laboratory standards NCCLS now CLSI [23]. The diameters of the inhibition zones produced around the test material were measured with a ruler and recorded in mm. Plant extracts that produced a zone of inhibition of 8–11 mm were said to be active. Those with zones above 11 mm were considered very active. Those with zone of inhibition below 8 mm were considered inactive [24]. The tests were replicated three times for each material.

### 2.5. Determination of Minimum Inhibitory Concentration

Minimum inhibitory concentration (MIC) was determined through the broth dilution technique. Different concentrations of the extracts were prepared and replicated three times. The extract concentrations were 100 mg/ml, 50 mg/ml, and 25 mg/ml. 5 ml of each concentration of the extract was poured aseptically into a sterile test tube. 1 ml of the toxigenic* A. flavus* (1 McFarland standard) was added. 1 ml of this mixture was poured aseptically into 5 ml of potato dextrose broth (serial dilution) [4]. All the tubes were incubated at 29°C for 72 hours. Observations were made for visible fungal growth. The lowest dilution without visible growth for each extract was regarded as the minimum inhibitory concentration

### 2.6. Treatment of Fungal Spores with Plant Extracts and Light

Concentrations of 450 mg/ml and 600 mg/ml of each plant extract were prepared as the working solutions. The 3 × 10^8^ CFU/ml McFarland solution was serially diluted up to a concentration of 3 × 10^2^ CFU/ml. 2 ml of this (3 × 10^2^ CFU/ml) spore suspension was added to 2 ml of each extract separately. The mixture was well shaken and treated with visible light spectrum at a range of 420 nm provided by a special lamp from Multiplex Display Fixture [19]. The maximum absorption range of the plant extracts was tested using a spectrophotometer and found to be 420 nm. Irradiation of the plant extracts which were the photosensitizers was done for 10, 20, and 40 minutes. Effects of light irradiated on the photosensitizer (plant extract) for varying time periods (10 mins, 20 mins, and 40 mins) were tested on the viability of spores of the toxigenic* A. flavus. *Controls experiments involved adding plant extracts to the conidia suspension without light treatments, reacting conidia without plant extracts with light and conidia without light and plant extracts as described by [19]. Each treatment was replicated three times.

100 ul of irradiated solution was transferred to PDA plates and incubated at 29°C. The control experiments were also treated in the same way. Colony forming units (CFUs) were counted after 72 hours of incubation to determine the viability of conidia.

### 2.7. Phytochemical Screening of Plant Extracts

The six organic and aqueous plant extracts obtained were subjected to phytochemical screening to determine the presence of bioactive agents like flavonoids, steroids, terpenoids, saponins, tannins, alkaloids, and glycosides. Plant extracts from the stock solution of 800 mg/ml were used for the phytochemical screening [4, 25].

### 2.8. Statistical Analysis

Data analysis was done using SPSS version 16. Data values were expressed as means ± standard error. Analysis of variance was used and when *F* was significant (*P* ≤ 0.05), comparison of means was done using Tukey's test.

## 3. Results

### 3.1. Effect of Organic and Aqueous Plant Extracts on Growth of Toxigenic* A. flavus*

The crude organic extracts of five out of the six plants tested exhibited antifungal activity against the growth of toxigenic strains of* A. flavus*. The aqueous extracts did not show significant (*P* ≤ 0.05) antifungal activity because all zones of inhibition diameter were below 8 mm.* Solanum aculeastrum* and* Syzygium cordatum* plant extracts at 600 mg/ml against* A. flavus* UONV017 had inhibition diameters that had no significance difference (*P* = 0.34 and *P* = 0.75) and hence compared favorably with the standard antifungal control Apron star (250 mg/ml) which is a class III Blue Active ingredient containing 20% thiamethoxam + 20% metalaxyl-M + 2% difenoconazole.* Solanum aculeastrum* organic extract had the highest antifungal activity followed by* Syzygium cordatum* against both strains of* A. flavus *(Tables 1 and 2). Apart from* S. aculeastrum* plant extracts against* A. flavus* UONV003 which had a higher inhibition diameter at 300 mg/ml (mean = 11.08 ± 0.67 mm) than at 450 mg/ml (mean = 11.00 ± 0.60 mm) and* P. africana* leaf extracts against* A. flavus* strain UONV017, which had a higher inhibition diameter at 450 mg/ml (mean = 9.25 ± 0.71 mm) than at 600 mg/ml (mean = 8.67 ± 0.69 mm), all the other extracts had the highest inhibitory activity at the highest concentration (600 mg/mL) and the lowest antifungal activity was at the lowest concentration (300 mg/ml) (Tables 1 and 2).

**Figure 1:**
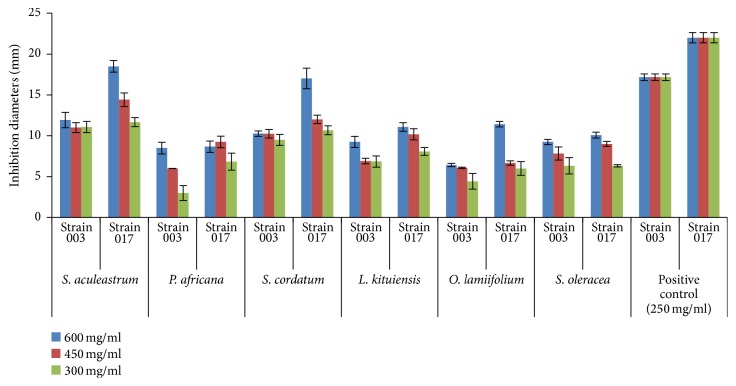
Comparison of inhibition of* A. flavus* UONV017 and that of* A. flavus* UONV003 by organic plant extracts at different concentrations.

Comparison of the activities of the organic plant extracts between both strains (UONV003 and UONV017) of* A. flavus* showed that* A. flavus* UONV003 had smaller inhibition diameters than* A. flavus* UONV017 at different concentrations (Figure 1).

Extracts of* S. aculeastrum* had the lowest minimum inhibitory concentration at 25 mg/ml against* A. flavus* (UONV017) and at 50 mg/ml against* A*.* flavus* UONV003.* Syzygium cordatum* had an MIC of 50 mg/ml on* A. flavus* UONV017 and 100 mg/ml on* A*.* flavus* UONV003.

### 3.2. Effect of Aqueous Plant Extracts and Photosensitization on* A. flavus* UONV017 at Different Concentrations and Different Timings

Interaction between aqueous plants extracts and visible light at 600 mg/ml was statistically significant (*F* = 55.80; DF = 18; *P* ≤ 0.05). Different plant extracts fungi suspensions had different counts of CFU after light treatment at varying time durations.* Solanum aculeastrum* (mean = 2 CFUs) had the lowest CFU reading at 40 minutes and hence was the most effective. Other than the controls,* S. oleracea* (mean = 13 CFUs) had the highest CFU reading at 10 minutes and hence was the least effective at 600 mg/ml. At 450 mg/ml, interaction between the aqueous extracts and light caused significant (*F* = 71.46; df = 18; *P* ≤ 0.05) reduction of CFUs.* Solanum aculeastrum* (mean = 3 CFUs) was the most effective with the lowest number of CFUs while* O. lamiifolium* (mean = 14 CFUs) was the least effective with the highest number of CFUs at 10 minutes.

Comparison of photosensitization activities within different time durations proved that treatments kept under light for the highest duration of time (40 minutes) had the lowest CFU counts, hence proving the highest inactivation of fungal spores. Treatments that were under light for the shortest time duration (10 minutes) exhibited a higher number of CFUs; samples with no light and no extract treatment had the highest CFU count (Table 3).

### 3.3. Effect of Aqueous Plant Extracts and Photosensitization on* A. flavus* (UONV003) at Different Concentrations and Different Timings

Statistically significant (*F* = 31.21; DF = 18; *P* ≤ 0.05) interaction existed between aqueous plants extracts and light in their activity against* A. flavus* strain UONV003 at 600 mg/ml.* Syzygium cordatum* (mean = 3 CFUs) had the lowest CFU reading at 40 minutes and hence was the most effective.* Lippia kituiensis* (mean = 16 CFUs) had the highest CFU reading at 10 minutes and hence was the least effective. At 450 mg/ml, interaction between aqueous extracts and light was also significant (*F* = 35.86; DF = 18; *P* ≤ 0.05).* Solanum aculeastrum* (mean = 3 CFUs) was the most effective while* L. kituiensis *(mean = 17 CFUs) was the least effective at 10 minutes (Table 4).

### 3.4. Effect of Organic Plant Extracts and Photosensitization on* A. flavus* (UONV017) at Different Concentrations and Different Timings

Organic extracts at both concentrations of 600 mg/ml displayed significant (*F* = 32.72; DF = 18; *P* ≤ 0.05) photosensitization activity.* Syzygium cordatum* (mean = 4 CFUs) had the lowest CFU reading at 40 minutes at a concentration of 600 mg/ml.* Ocimum lamiifolium* (mean = 19 CFUs) had the highest CFU reading at 10 minutes at 600 mg/ml. At 450 mg/ml, there was also significant (*F* = 23.615; DF = 18; *P* ≤ 0.05) photosensitization activity.* Syzygium cordatum* (mean = 7 CFUs) was the most effective at 40 minutes (Table 5).

### 3.5. Effect of Aqueous Plant Extracts and Photosensitization on* A. flavus* (UONV003) at Different Concentrations and Different Timings

Interaction between organic plants extracts and light was statistically significant (*F* = 32.97; DF = 18; *P* ≤ 0.05) at a concentration of 600 mg/ml.* Solanum aculeastrum* (mean = 9 CFUs) had the lowest CFU reading at 40 minutes.* Lippia kituiensis* (mean = 23 CFUs) had the highest CFU reading at 10 minutes and hence was the least effective at 600 mg/ml. At 450 mg/ml, organic extracts exhibited significant (*F* = 19.39; DF = 18; *P* ≤ 0.05) photosensitization activity.* Solanum aculeastrum *(mean = 9 CFUs) was still the most effective at 40 minutes (Table 6).

Comparison of photosensitization effects between aqueous and organic extracts proved that both extracts were effective against toxigenic* A. flavus* (UONV017 and UONV003) because there was significant reduction of CFU in comparison with the controls. Aqueous extracts had a lower number of CFUs than the organic extracts. The higher concentration (600 mg/ml) had greater reduction of CFU compared to the 450 mg/ml (Table 6).

### 3.6. Phytochemical Screening of Selected Medicinal Plant Extracts

Six different bioactive compounds, namely, saponins, flavonoids, terpenoids, tannins, alkaloids, and glycosides, were found in the plant extracts during this study.* Spinacia oleracea *plant extracts had the highest percentage (21.6), followed by* S. aculeastrum* (19.6%), while* Prunus africana* had the lowest percentage (9.8) of bioactive compounds. Flavonoids had the highest frequency (21.6%) while terpenoids and steroids had the lowest frequency (9.8%). Organic extracts had a higher frequency (60.8%) of bioactive compounds compared to aqueous extracts (39.2%) (Table 7).

## 4. Discussions

This study revealed that the selected medicinal plant extracts had antifungal activity against toxigenic* A. flavus. *Five out of the six organic plants extracts assayed indicated antifungal activity which varied depending on concentration and plant species. At 600 mg/ml, the antifungal activity of* S. aculeastrum* and* S. cordatum* had no significant difference with the standard antifungal control Apron star. This reveals the significance of crude plant extracts in lowering risk of toxigenic* A. flavus,* hence lowering aflatoxin contamination [26].

Organic extracts exhibited significant antifungal activity while the aqueous extracts did not. The organic extracts had a higher percentage of bioactive compounds (60.8%) than the aqueous extracts (39.2%). Different solvents have varying levels of solubility for different bioactive compounds. The reason for this sequence in activity may be that bioactive compounds that cause the antimicrobial activity dissolve easily in organic compared to aqueous solvents [27]. This supports earlier findings that organic leaf extracts had stronger antimicrobial activity compared to aqueous extracts [28]. The antifungal activity was directly proportional to the concentration of the plant extracts; the highest concentration (600 mg/ml) had the largest inhibition diameters while the lowest concentration (300 mg/ml) had the smallest inhibition diameters. This could be explained by the fact that the high concentration contains a higher percentage of the bioactive compounds. This observation parallels findings in a study carried out by Mahmoud et al. [29] and Kiswii et al. [4]. Phytochemical screening indicated presence of tannins, flavonoids, alkaloids, glycosides, steroids, and saponins in varying proportions in the plant extracts. The antifungal efficacy of plant extracts in this study could be associated with the bioactive compounds indicated by the phytochemical analysis. Organic fruit extracts of* S. aculeastrum* had the best antifungal activity against both strains of toxigenic* A. flavus. Solanum aculeastrum* in our study contained ten bioactive compounds which is 19.6% according to the phytochemical screening. These could be the possible cause of the antifungal activity. The glycosides, alkaloids, and saponins in* S. aculeastrum* fruits have been associated with anticancer (skin and cervix), anti-inflammatory, and anticholesterol activities [30]. This agrees with the results of [31] where organic and aqueous fruit extracts of* S. aculeastrum* were tested against fungi and bacteria and they had good antimicrobial activity although antifungal activity caused by organic extracts was higher than that caused by aqueous extracts.* Syzygium cordatum* bark extracts had significant antifungal activity against both* A. flavus* strains. This agrees with [32] where* S. cordatum* bark (organic extract) exhibited antifungal activity. In this study,* S. cordatum* contains nine bioactive compounds which are 17.6%. These as supported by [33] were the causes of antifungal activity.* Syzygium cordatum* at a concentration of 450 mg/ml had higher inhibition diameter compared to that of 600 mg/ml. This could have been caused by the thickness of the higher concentration which interfered with diffusion of the extract and hence a lower diameter of inhibition.

Organic extracts of* P. africana, L. kituiensis, S. oleracea,* and* O. lamiifolium* had antifungal activity against both strains of* A. flavus*. They all had varying levels of bioactive compounds which were attributed to the varying levels of antifungal activity and this justifies the use of these plant extracts in ethnomedicine. The percentage of bioactive compounds (9.8%) in* P. africana* was the lowest and this could be attributed to the low antifungal activity by this extract. Organic extracts of* P. africana* against* A. flavus* UONV017 had higher antifungal activity at 450 mg/ml than at 600 mg/ml. This could have been caused by thickness in the higher concentration which interfered with diffusion.* Spinacia oleracea* extracts had the highest percentage of bioactive compounds (21.6%). Despite having the highest percentage of bioactive compounds, it did not prove the highest level of antifungal activity. This could be attributed to presence of the bioactive compounds in small amounts. Studies by [28, 34, 35] have proved that bioactive compounds in these plant extracts cause them to have antifungal activity.

The bioactive compounds detected in this study have been shown to cause antimicrobial activities in other studies through various mechanisms [27, 36–38]. Increase in the concentration of bioactive compounds increases the antifungal activity as reported by [39]. This supports the increase in antifungal activity caused by increase in plant extract concentration in our study.

*Solanum aculeastrum* that had the highest antifungal activity recorded the lowest MIC values for both strains. The low MIC could be attributed to the enhanced antifungal activity. Kiswii et al. [4] found out that the extracts with the highest antifungal activity had the lowest MIC value.

Strain UONV003 of* A. flavus* proved to be more resistant compared to* A. flavus* UONV017 in terms of inhibition diameters and also MIC though both are toxigenic strains. This could be explained by the fact that pathogenicity varies between different strains of* A. flavus* and strains with higher pathogenicity may exhibit higher resistance. This parallels a study that was carried out to test pathogenicity and toxigenicity of ground nut* A. flavus* strains. Results showed that there was variation in pathogenicity within the same level of toxigenic strains [40].

This study found that both aqueous and organic plant extracts in combination with light inactivated* A. flavus* spores, though the former were more effective.* Syzygium cordatum* which had significant inhibition even in the aqueous form against both strains have been reported to have several antimicrobial abilities [32].* Solanum aculeastrum* was the second most effective against both strains in the organic form and the most effective in the aqueous form against both strains.* Solanum aculeastrum* possess several antimicrobial activities and of significance to this study; the fruit extracts possess antifungal activity against* Aspergillus *spp. [30].

Both aqueous and organic extracts of the other four plants,* O. lamiifolium, S. oleracea, P*.* africana,* and* L. kituiensis,* had significant reduction of spores as they had low CFU counts compared to the controls against both strains. All the named plants have been reported to possess several antimicrobial activities [28, 41–43]. The higher CFU count in the three controls is an indicator that the visible light plant extract combination was the major cause of conidia inactivation and not light alone or plant extracts alone.

The photo degrading effect of plant extracts in this study is supported by a study where viability* Penicillium digitatum *conidia was tested using blue light and a dye (ERY) acting as a photosensitizer. Blue light alone or the photosensitizer alone did not reduce the viability of the nongerminated conidia as compared to the conidia viability control. The control was comprised of no light and no photosensitizer treatment. However, nongerminated conidia treated with light and photosensitizer significantly reduced colony forming units (CFUs) by 40 and 70% with blue light of 80 and 100 J/cm^2^, respectively, compared to control [44]. In another study, plant extracts that affect the central nervous system were tested for photoprotection and photosensitization. They were tested at wavelengths ranging from 280 nm to 436 nm. The plants showed photo protection effect at low concentration and photosensitization effects at the higher concentrations [45]. This also parallels this study where the photosensitization effect was directly proportional to the concentration of the plant extracts. This is explained by the fact that higher concentrations have high number of bioactive compounds which are reactivated by light to inactivate toxigenic microbes.

Photosensitization effect is attributed to the contents of the plant extracts which have strong absorption at high wavelength range [45]. Disinfection of water using the solar energy and plant extracts has been proven. Photodynamic activity was attributed to the presence of quinines and anthraquinones which generate singlet oxygen killing the microorganisms in the water. Two ml of plant extract per one litre of the polluted water was exposed to the solar energy for one hour to allow complete inactivation of the coli forms [46]. This parallels our study where plant extracts in combination with toxigenic molecules are exposed to light for a specific duration leading to reduced fungal growth.

In this study, the time of exposure of the* A. flavus* and plant extract under light was indirectly proportional to the CFU counts; this indicates that more light exposure led to more spore knockout. This parallels a study by [19] where increase in light dose increased the rate of spore knockout. In this study also, spore reduction was higher at the higher plant extract concentration of 600 mg/ml compared to the 450 mg/ml. In the study by [19], rate of spore knockout increased with the extract concentrations up to a certain level where the rate of spore knockout decreased with the highest concentration. This was attributed to the high fluid turbidity which may have caused reduced light penetration and transmission. In this study, therefore, the extract concentrations were at a suitable turbidity which did not cause much inhibition of light penetration and inhibition.

Both aqueous and organic extracts were effective photosensitizers though aqueous extracts had a greater reduction of spores than the organic extracts. This is supported by a study by [19] where both organic and aqueous extracts were effective though the aqueous extracts had better activity.

Extracts used in this study have not been tested for antifungal activity and photosensitization against toxigenic* A. flavus* in another study. The findings of this study, therefore, could fulfill the need of new antifungal structure due to unavailability of effective antifungal agents against toxigenic* A. flavus*, resistance of the fungi to the available methods, and the shortcomings of these methods [46]. The plants in the selected region were correlated with the biodiversity of the region and they are accessible for research and new developments. Plant extracts are of key importance because bioactive compounds in higher plants are biodegradable and selectively toxic.

## 5. Conclusion and Recommendations

In this study, the medicinal plant extracts were found effective against conidia of toxigenic* A. flavus. *Organic extracts had greater antifungal activity. This makes a good background for research on aflatoxins because aflatoxins also dissolve better in organic extracts compared to aqueous ones. The extracts were found to contain bioactive compounds which were the specific causes of antifungal activity. Isolation of these useful bioactive compounds using the guidance of phytochemical results should be done. The bioactive compounds should be produced in larger quantities for use in the control of the toxigenic fungi. The consumable plant extract* Spinacia oleracea* in this study can act as a good candidate to treat food and feed which can then be dried under sunlight before storage leading to inactivation and death of the toxigenic* A. flavus* conidia. This is a viable technique to control aflatoxin contamination in storage because consumption of the dead* A. flavus* has no health implications. This could be applied to all the plant extracts in this study after a toxicity test to ensure safety.

## Figures and Tables

**Table 1:** Effect of different plant extracts on growth of *A. flavus* strain UONV003 at different concentrations.

Plants	Inhibition zones (mm) 600 mg/ml	Inhibition zones (mm) 450 mg/ml	Inhibition zones (mm) 300 mg/ml
*S. aculeastrum*	11.92 ± 0.94^ab^	11.00 ± 0.60^b^	11.08 ± 0.67^b^
*S. cordatum*	10.27 ± 0.32^bc^	10.25 ± 0.52^b^	9.50 ± 0.68^bc^
*L. kituiensis*	9.25 ± 0.70^c^	6.92 ± 0.34^c^	6.58 ± 0.70^cd^
*P. africana*	8.50 ± 0.72^cd^	6.00 ± 0.01^c^	3.00 ± 0.90^e^
*O. lamiifolium*	6.42 ± 0.20^d^	6.08 ± 0.08^c^	4.42 ± 0.96^de^
*S. oleracea*	9.25 ± 0.31^c^	7.83 ± 0.79^c^	6.33 ± 0.99^c–e^
Positive control 250 mg/ml	17.17 ± 0.40^a^	17.17 ± 0.40^a^	17.17 ± 0.40^a^
Sig *P* < 0.05	0.00	0.00	0.00

Numbers are means of twelve replications. One-way Annova was used for analysis and means were separated by Tukey's test. Numbers followed by the same letters in the same column are not significantly different (*P* < 0.05).

**Table 2:** Effect of different plant extracts against growth of *A. flavus* strain UONV017 at different concentrations.

Plants	Inhibition zones (mm) 600 mg/ml	Inhibition zones (mm) 450 mg/ml	Inhibition zones (mm) 300 mg/ml
*S. aculeastrum*	18.50 ± 0.71^a^	14.42 ± 0.83^b^	11.67 ± 0.54^b^
*S. cordatum*	17.00 ± 1.26^a^	12.00 ± 0.52^bc^	10.67 ± 0.54^bc^
*L. kituiensis*	11.08 ± 0.53^b^	10.17 ± 0.68^cd^	8.08 ± 0.47^cd^
*P. africana*	8.67 ± 0.69^b^	9.25 ± 0.71^d^	6.83 ± 1.04^d^
*O. lamiifolium*	11.42 ± 0.34^b^	6.67 ± 0.26^e^	6.00 ± 0.83^d^
*S. oleracea*	10.08 ± 0.36^b^	9.00 ± 0.30^de^	6.33 ± 0.14^d^
Positive control 250 mg/ml	22.00 ± 0.63^a^	22.00 ± 0.63^a^	22.00 ± 0.63^a^
(Sig *P* < 0.05)	0.00	0.00	0.00

Numbers are means of twelve replications. One-way Annova was used for analysis and means were separated by Tukey's test. Numbers followed by the same letters in the same column are not significantly different (*P* < 0.05).

**Table 3:** Effect of aqueous plant extracts and photosensitization on *Aspergillus flavus* (UONV017).

Plants	Colony forming units at 600 mg/ml				Colony forming units at 450 mg/ml			
	10 (min)	20 (min)	40 (min)	0 minutes (ctrl)	10 (min)	20 (min)	40 (min)	0 minutes (ctrl)
*S. aculeastrum*	4.00 ± 1.29^b^	4.00 ± 1.29^ab^	2.00 ± 1.29^a^	35 ± 1.29^c^	4.00 ± 1.12^b^	4.00 ± 1.12^ab^	3.00 ± 1.12^a^	35 ± 1.12^c^
*P. africana*	5.00 ± 1.29^b^	4.00 ± 1.29^ab^	3.00 ± 1.29^a^	71 ± 1.29^c^	6.00 ± 1.12^b^	5.00 ± 1.12^ab^	5.00 ± 1.12^a^	71 ± 1.12^c^
*S. cordatum*	5.00 ± 1.29^b^	4.00 ± 1.29^ab^	4.00 ± 1.29^a^	41 ± 1.29^c^	5.00 ± 1.12^b^	4.00 ± 1.12^ab^	4.00 ± 1.12^a^	41 ± 1.12^c^
*L. kituiensis*	11.00 ± 1.29^b^	8.00 ± 1.29^ab^	7.00 ± 1.29^a^	41 ± 1.29^c^	13.00 ± 1.12^b^	11.00 ± 1.12^ab^	10.00 ± 1.12^a^	41 ± 1.12^c^
*O. lamiifolium*	12.00 ± 1.29^b^	8.00 ± 1.29^ab^	6.00 ± 1.29^a^	62 ± 1.29^c^	14.00 ± 1.12^b^	11.00 ± 1.12^ab^	10.00 ± 1.12^a^	62 ± 1.12^c^
*S. oleracea*	13.00 ± 1.29^b^	10.00 ± 1.29^ab^	7.00 ± 1.29^a^	54 ± 1.29^c^	12.00 ± 1.12^b^	11.00 ± 1.12^b^	10.00 ± 1.12^a^	54 ± 1.12^c^
0 extracts (ctrl)	76.00 ± 1.29^ab^	73.00 ± 1.29^b^	73.00 ± 1.29^b^	80.00 ± 1.29^a^	76.00 ± 1.12^ab^	73.00 ± 1.12^b^	73.00 ± 1.12^b^	80.00 ± 1.12^a^

Numbers are means of three replications. Two-way Annova was used for analysis and means were separated by Tukey's test. Numbers followed by the same letters in the same row within each concentration are not significantly different (*P* < 0.05).

**Table 4:** Effect of aqueous plant extracts and photosensitization on *Aspergillus flavus* UONV003.

Plants	Colony forming units at 600 mg/ml				Colony forming units at 450 mg/ml			
	10 (min)	20 (min)	40 (min)	0 minutes (ctrl)	10 (min)	20 (min)	40 (min)	0 minutes (ctrl)
*S. aculeastrum*	5.00 ± 1.70^b^	4.00 ± 1.70^ab^	4.00 ± 1.70^a^	43 ± 1.70^c^	5.00 ± 1.60^b^	4.00 ± 1.60^ab^	4.00 ± 1.60^a^	43 ± 1.60^c^
*P. africana*	6.00 ± 1.70^b^	5.00 ± 1.70^ab^	4.00 ± 1.70^a^	72 ± 1.70^c^	5.00 ± 1.60^b^	4.00 ± 1.60^ab^	4.00 ± 1.60^a^	72 ± 1.60^c^
*S. cordatum*	5.00 ± 1.70^b^	4.00 ± 1.70^ab^	3.00 ± 1.70^a^	34 ± 1.70^c^	5.00 ± 1.60^b^	4.00 ± 1.60^ab^	3.00 ± 1.60^a^	34 ± 1.60^c^
*L. kituiensis*	16.00 ± 1.70^b^	12.00 ± 1.70^ab^	8.00 ± 1.70^a^	44 ± 1.70^c^	17.00 ± 1.60^b^	14.00 ± 1.60^ab^	12.00 ± 1.60^a^	44 ± 1.60^c^
*O. lamiifolium*	13.00 ± 1.70^b^	11.00 ± 1.70^ab^	8.00 ± 1.70^a^	56 ± 1.70^c^	15.00 ± 1.60^b^	11.00 ± 1.60^ab^	10.00 ± 1.60^a^	56 ± 1.60^c^
*S. oleracea*	14.00 ± 1.70^b^	14.00 ± 1.70^ab^	11.00 ± 1.70^a^	58 ± 1.70^c^	16.00 ± 1.60^b^	13.00 ± 1.60^ab^	10.00 ± 1.60^a^	58 ± 1.60^c^
0 extracts (ctrl)	76.00 ± 1.70^a^	76.00 ± 1.70^a^	74.00 ± 1.70^a^	81.00 ± 1.70^a^	76.00 ± 1.60^a^	76.00 ± 1.60^a^	74.00 ± 1.60^a^	81.00 ± 1.60^a^

Numbers are means of three replications. Two-way Annova was used for analysis and means were separated by Tukey's test. Numbers followed by the same letters in the same row within each concentration are not significantly different (*P* < 0.05).

**Table 5:** Effect of organic plant extracts and photosensitization on *Aspergillus flavus* (UONV017).

Plants	Colony forming units at 600 mg/ml				Colony forming units at 450 mg/ml			
	10 (min)	20 (min)	40 (min)	0 minutes (ctrl)	10 (min)	20 (min)	40 (min)	0 minutes (ctrl)
*S. aculeastrum*	11.00 ± 1.7^b^	11.00 ± 1.7^a^	8.00 ± 1.7^a^	62 ± 1.71^c^	24.00 ± 1.73^b^	12.00 ± 1.73^a^	9.00 ± 1.7^a^	62 ± 1.73^c^
*P. africana*	10.00 ± 1.7^b^	10.00 ± 1.7^a^	8.00 ± 1.7^a^	76 ± 1.71^c^	41.00 ± 1.73^b^	23.00 ± 1.73^a^	14.00 ± 1.73^a^	76 ± 1.73^c^
*S. cordatum*	10.00 ± 1.7^b^	8.00 ± 1.71^a^	4.00 ± 1.7^a^	51 ± 1.71^c^	11.00 ± 1.73^b^	8.00 ± 1.73^a^	7.00 ± 1.73^a^	51 ± 1.73^c^
*L. kituiensis*	16.00 ± 1.7^b^	12.00 ± 1.7^a^	8.00 ± 1.7^a^	44 ± 1.71^c^	22.00 ± 1.73^b^	12.00 ± 1.73^a^	11.00 ± 1.73^a^	48 ± 1.73^c^
*O. lamiifolium*	19.00 ± 1.7^b^	12.00 ± 1.7^a^	9.00 ± 1.7^a^	48 ± 1.71^c^	23.00 ± 1.73^b^	16.00 ± 1.73^a^	14.00 ± 1.73^a^	72 ± 1.73^c^
*S. oleracea*	11.00 ± 1.7^b^	10.00 ± 1.7^a^	9.00 ± 1.7^a^	47 ± 1.71^c^	14.00 ± 1.73^b^	12.00 ± 1.73^a^	9.00 ± 1.73^a^	47 ± 1.73^c^
0 extracts (ctrl)	76.00 ± 1.7^ab^	73.00 ± 1.7^b^	73.00 ± 1.1^b^	80.00 ± 1.71^a^	76.00 ± 1.73^b^	73.00 ± 1.73^b^	73.00 ± 1.73^b^	80.00 ± 1.73^ab^

Numbers are means of three replications. Two-way Annova was used for analysis and means were separated by Tukey's test. Numbers followed by the same letters in the same row within each concentration are not significantly different (*P* < 0.05).

**Table 6:** Effect of organic plant extracts and photosensitization on *Aspergillus flavus* UONV003.

Plants	Colony forming units at 600 mg/ml				Colony forming units at 450 mg/ml			
	10 (min)	20 (min)	40 (min)	0 minutes (ctrl)	10 (min)	20 (min)	40 (min)	0 minutes (ctrl)
*S. aculeastrum*	12.00 ± 1.7^b^	12.00 ± 1.76^b^	9.00 ± 1.76^a^	69 ± 1.76^c^	26.00 ± 2.22^b^	13.00 ± 2.22^a^	9.00 ± 2.22^a^	69 ± 2.22^c^
*P. africana*	18.00 ± 1.7^b^	17.00 ± 1.76^b^	11.00 ± 1.7^a^	77 ± 1.76^c^	34.00 ± 2.22^b^	20.00 ± 2.22^a^	15.00 ± 2.2^a^	77 ± 2.22^c^
*S. cordatum*	15.00 ± 1.7^b^	14.00 ± 1.76^b^	10.00 ± 1.7^a^	60 ± 1.76^c^	18.00 ± 2.22^b^	15.00 ± 2.22^a^	12.00 ± 2.2^a^	60 ± 2.22^c^
*L. kituiensis*	23.00 ± 1.7^b^	12.00 ± 1.76^b^	14.00 ± 1.7^a^	64 ± 1.76^c^	27.00 ± 2.22^b^	13.00 ± 2.22^a^	11.00 ± 2.2^a^	74 ± 2.22^c^
*O. lamiifolium*	12.00 ± 1.7^b^	10.00 ± 1.76^b^	11.00 ± 1.7^a^	74 ± 1.76^c^	13.00 ± 2.22^b^	10.00 ± 2.22^a^	10.00 ± 2.2^a^	52 ± 2.22^c^
*S. oleracea*	12.00 ± 1.7^b^	12.00 ± 1.7^b^	10.00 ± 1.7^a^	52 ± 1.76^c^	11.00 ± 2.22^b^	11.00 ± 2.22^a^	10.00 ± 2.2^a^	47 ± 2.22^c^
0 extracts (ctrl)	76.00 ± 1.7^a^	76.00 ± 1.76^a^	74.00 ± 1.7^a^	81.00 ± 1.7^a^	76.00 ± 2.22^a^	76.00 ± 2.22^a^	74.00 ± 2.2^a^	81.00 ± 2.2^a^

Numbers are means of three replications. Two-way Annova was used for analysis and means were separated by Tukey's test. Numbers followed by the same letters in the same row within each concentration are not significantly different (*P* < 0.05).

**Table 7:** Frequency of bioactive compounds in plant extracts.

	Frequency	Percent	Cumulative percent
Plants			
* Prunus africana*	5	9.8	9.8
* Lippia kituiensis*	7	13.7	23.5
* Solanum aculeastrum*	10	19.6	43.1
* Syzygium cordatum*	9	17.6	60.8
* Spinacia oleracea*	11	21.6	82.4
* Ocimum lamiifolium*	9	17.6	100
Total	**51**	**100**	
Bioactive compounds			
Saponins	8	15.7	15.7
Tannins	9	17.6	33.3
Flavonoids	11	21.6	54.9
Alkaloids	10	1.96	74.5
Glycosides	8	15.7	90.2
Terpenoids and steroids	5	9.8	100
Total	**51**	**100**	
Formulation			
Organic	31	60.8	60.8
Aqueous	20	39.2	100
Total	**51**	**100**	
